# A Dimensional Approach to PTSD After Drug‐Facilitated Sexual Assault: Impact on Sexual Functioning

**DOI:** 10.1155/da/6023533

**Published:** 2026-10-31

**Authors:** Marta Lizarán, Adrián Torres-Villasante, Julia Andreu-Martínez, Nadina Gómez-Merino, Javier Sánchez-Ruiz, Javier Plumed-Domingo, Alba Moreno-Giménez, Ana García-Blanco

**Affiliations:** ^1^ Department of Psychiatry and Clinical Psychology La Fe University and Polytechnic Hospital Avenida Fernando Abril Martorell 106 Valencia 46026 Spain hospital-lafe.com; ^2^ Mental Health Research Group Health Research Institute La Fe Avenida Fernando Abril Martorell 106 Valencia 46026 Spain iislafe.es; ^3^ Department of Personality Evaluation and Psychological Treatment Faculty of Psychology University of Valencia Avenida Blasco Ibáñez, 21 Valencia 46010 Spain uv.es; ^4^ ERI-Lectura Faculty of Psychology University of Valencia Avenida Blasco Ibáñez, 21 Valencia 46010 Spain uv.es; ^5^ Department of Developmental and Educational Psychology Faculty of Psychology University of Valencia Avenida Blasco Ibáñez, 21 Valencia 46010 Spain uv.es; ^6^ Department of Methodology of Behavioral Sciences Faculty of Psychology University of Valencia Avenida Blasco Ibáñez, 21 Valencia 46010 Spain uv.es

**Keywords:** DFSA, PTSD, sexual assault, sexual functioning, sexual trauma

## Abstract

Sexual assault (SA) is linked to both posttraumatic stress disorder (PTSD) symptoms and sexual functioning (SF) difficulties, but the psychological consequences of drug‐facilitated SA (DFSA) remain insufficiently characterized. This study primarily compared PTSD symptom profiles between DFSA and non‐DFSA survivors, while secondarily examining SF deterioration and exploring associations between PTSD symptom profiles and SF impairment. Thirty‐nine women who experienced an isolated SA within the past year (20 non‐DFSA, 19 DFSA) and 35 nontraumatized controls were recruited. Participants with prior SA, trauma history, or previous psychiatric disorders were excluded. PTSD symptoms were assessed only in the survivor groups. Although both survivor groups met PTSD criteria at similar rates, DFSA survivors exhibited fewer and less severe symptoms, particularly in reexperiencing, distress, avoidance, and dissociation. Both survivor groups reported SF deterioration after the SA compared with pre‐assault functioning. In exploratory linear regression analyses, greater hyperarousal severity, but not negative alterations in cognition and mood, was associated with greater SF deterioration. These findings suggest that DFSA is characterized by a distinct profile of PTSD symptoms despite comparable diagnostic rates, emphasizing the importance of moving beyond categorical PTSD diagnoses toward symptom‐informed clinical assessment. Such an approach may facilitate more personalized trauma‐informed interventions. The exploratory association between hyperarousal and SF deterioration provides preliminary evidence for a potential therapeutic target that warrants confirmation in larger prospective studies.

## 1. Introduction

Sexual assault (SA), defined as unwanted, nonconsensual sexual contact achieved through force, incapacitation, and/or coercion, is one of the most prevalent and severe forms of interpersonal trauma. Global estimates indicate that approximately one in three women experience sexual violence during their lifetime, making SA a major public health concern with profound psychological and physical consequences [1, 2]. Beyond its well‐established impact on sexual functioning (SF) [3, 4], SA is widely regarded as the interpersonal traumatic event most strongly linked to posttraumatic stress disorder (PTSD), often involving reexperiencing symptoms such as flashbacks or nightmares related to trauma recall [5]. However, not all SA survivors have a vivid trauma experience. In drug‐ or alcohol‐facilitated SA (DFSA), defined as an SA in which the survivor is incapacitated or unable to consent due to the voluntary or involuntary intake of a substance [6], substance‐induced alterations in consciousness and memory may disrupt the encoding and consolidation of the traumatic experience. Despite being a common and increasingly reported subtype of SA [7], DFSA remains under‐researched [8], particularly regarding its impact on PTSD symptom presentation and postassault SF.

Given its particular characteristics, DFSA may involve partially distinct psychological pathways to PTSD. Substance‐induced alterations in consciousness may disrupt trauma encoding and autobiographical memory integration, hindering the construction of a coherent trauma narrative [6, 7]. Consequently, although SA survivors (with or without DF) may develop PTSD, DFSA may be associated with a different pattern of symptom expression, characterized by greater confusion, self‐blame, and loss of control, rather than fear‐ and coercion‐related appraisals more typically described following non‐DFSA [9–11]. According to this possibility, the empirical evidence remains heterogeneous and inconclusive. Some studies have identified DFSA as a risk factor for greater PTSD severity [12, 13], whereas others have reported attenuated PTSD symptoms [10, 14]. Conversely, several studies have found no differences in overall PTSD severity between DFSA and non‐DFSA survivors [7, 8, 15].

These discrepant findings are likely attributable, at least in part, to methodological differences across studies. Most previous investigations have relied on community samples with broad age ranges [10, 12–14] and heterogeneous trauma histories, frequently including survivors with childhood sexual abuse, prior revictimization, multiple traumatic experiences, or pre‐existing psychiatric disorders [10, 12, 13], all of which are well‐established determinants of PTSD severity [16–18]. Importantly, comparisons between DFSA and non‐DFSA survivors are further complicated by systematic differences in assault characteristics. DFSA typically involves a single assault with penetration, most commonly affecting young women in nightlife settings (see [19] for a systematic review). By contrast, non‐DFSA encompasses a broader spectrum of assault experiences, including assaults involving physical force or coercion, repeated victimization, and assaults more frequently perpetrated by an acquaintance [20]. It is also more commonly associated with a history of childhood sexual abuse [21]. Because these assault‐related characteristics are themselves associated with PTSD severity and recovery trajectories, comparisons between DFSA and non‐DFSA require clinically homogeneous samples that are closely matched on both pre‐assault vulnerability factors and assault‐related variables. Otherwise, differences attributed to drug facilitation may instead reflect variations in participants’ clinical background or in the nature of the assault itself.

Another important methodological consideration of the existing literature is its predominant reliance on categorical PTSD diagnoses or overall symptom severity. However, PTSD is a multidimensional disorder, and specific symptom dimensions may vary according to the characteristics of the traumatic event. Consistent with this perspective, Fields et al. [7] reported that, despite comparable overall PTSD severity, DFSA survivors attending a specialized trauma clinic exhibited fewer dissociative and depersonalization symptoms than non‐DFSA survivors. Together, these findings suggest that examining PTSD symptom profiles may provide a more nuanced characterization of posttraumatic responses following DFSA.

Although PTSD has received relative attention in DFSA research, SF represents another major domain of postassault recovery. Survivors frequently report reduced sexual desire, difficulties with arousal, lubrication, and orgasm, lower sexual satisfaction, and sexual avoidance [3, 4, 22–24]. Compared with survivors of nonsexual traumatic events, women exposed to SA are substantially more likely to experience persistent sexual difficulties [25–31], suggesting that sexual trauma has unique consequences for later sexual well‐being. Additionally, sexual trauma may alter the emotional meaning associated with sexual activity, linking sexuality with fear, coercion, guilt, or self‐recrimination, which may contribute to subsequent SF difficulties [9]. However, despite robust evidence linking SA to impaired SF, little is known about whether the magnitude or pattern of these impairments differs between DFSA and non‐DFSA survivors.

PTSD has been proposed as a key mechanism linking SA and subsequent SF impairment [32, 33]. Clinical studies indicate that survivors meeting diagnostic criteria for PTSD experience greater sexual difficulties than those without PTSD, even after accounting for previous psychiatric disorders and earlier sexual victimization [3]. Nevertheless, most studies have conceptualized PTSD categorically, overlooking the possibility that individual symptom dimensions may differentially contribute to SF. The limited evidence available supports this hypothesis. For instance, hyperarousal, given the increased vigilance and physiological tension, has been linked to poorer sexual satisfaction [33–35], whereas reexperiencing symptoms have been associated with orgasm difficulties [22]. Together, although sexual dysfunction following SA has been widely documented, little is known about whether these impairments differ between DFSA and non‐DFSA survivors or whether distinct PTSD symptom dimensions are differentially associated with postassault SF.

To address these gaps, the present study recruited a clinically homogeneous sample of women who had experienced a single episode of SA within the previous year, with or without drug facilitation, while excluding survivors with previous sexual victimization, other traumatic experiences, or pre‐existing psychiatric disorders. A control group without a history of trauma or SA, matched on sex, age, ethnicity, and educational level, was included to provide a reference for baseline SF. The primary objective of this study was to characterize and compare PTSD symptom severity and symptom clusters between DFSA and non‐DFSA survivors. The secondary objective was to examine changes in SF following the assault and assess whether the magnitude of these changes differed across groups. Finally, exploratory analyses were conducted to identify which PTSD symptom clusters were most strongly associated with SF impairment among survivors. The study hypotheses are as follows. First, both SA groups were expected to meet PTSD criteria at comparable rates. However, DFSA survivors were expected to show lower reexperiencing, avoidance, and dissociative symptoms due to the fragmented trauma narrative and reduced perceptual awareness commonly associated with DFSA (see [7]). Second, both SA groups were expected to exhibit a decline in SF postassault, as this is a well‐established consequence after a sexual trauma [24]. Finally, exploratory analyses were conducted to examine whether symptom dimensions related to negative alterations in cognition, mood, and hyperarousal were associated with SF impairment among SA survivors, given their potential role in shaping threat perception, emotional distress, and difficulties during intimate experiences (see [3, 33–35]).

## 2. Methods

### 2.1. Participants

Participants were recruited between October 2022 and October 2024 from a public outpatient mental health center specializing in sexual trauma, which serves as the reference center for the entire region, covering a catchment area of ~5.4 million people. Clinical records of 400 consecutive patients were screened to identify potentially eligible participants. To ensure the recency of the traumatic event and to enhance the validity and clinical relevance of symptom assessment specifically related to sexual trauma, only women whose assault had occurred within the previous 12 months were considered eligible. Following the initial screening, 133 adult civilian women met the criterion.

The exclusion criteria were as follows: (i) being under 18 years old, (ii) having experienced childhood sexual abuse or any prior SA (to avoid the influence of early traumatic experiences on PTSD symptoms and SF, as these are well‐established confounding factors), (iii) exposure to other traumatic events (e.g., environmental disasters, kidnaping, murder of a family member, death threats), and (iv) having a previous psychiatric diagnosis. After applying these criteria, 39 survivors of a recent, isolated SA were recruited and categorized into two groups based on clinical records completed by trained professionals and supplemented with survivors’ self‐reports during clinical assessments: (i) survivors of SA involving physical force or coercion without substances (non‐DFSA; *N* = 19) and (ii) survivors of DFSA, meaning the survivor reported being under the influence of substances (administered voluntarily or involuntarily) that limited their capacity to resist or recall the event (*N* = 19). Additionally, a control group of 39 adult women was recruited via community advertisements. The control group consisted of women with no history of SA or other traumatic events, matched on age, nationality, and education level to the survivors, serving as a reference for SF and psychological symptoms in a nontraumatized population. After applying the same exclusion criteria—along with the additional requirement that none had experienced SA—35 cases were included. Figure 1 illustrates the participant recruitment and selection process.

**Figure 1:**
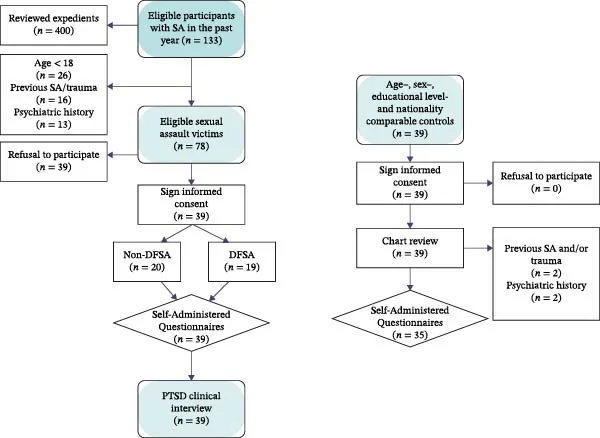
Sample recruitment flowchart.

Because the study addressed three distinct analytical objectives, separate a priori power analyses were conducted using 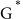
Power (version 3.1). For the primary PTSD analyses, power was estimated for the main between‐group MANOVA comparing two assault groups (DFSA vs. non‐DFSA) across six CAPS‐5 symptom dimensions. Assuming *α* = 0.05, power = 0.80, and a plausible moderate‐to‐large multivariate effect size range (*f* ^2^(*V*) = 0.08–0.12), the estimated required sample size ranged from ~35 to 52 participants. The final survivor sample included 39 participants. For the secondary analyses of SF, power was estimated for a mixed repeated‐measures ANOVA (within–between interaction) including two groups (DFSA and non‐DFSA) and two time points (before vs. after the assault). Assuming *α* = 0.05, power = 0.80, and a plausible medium‐to‐large effect size range (*f* = 0.25–0.35), the estimated required sample size was 19–34 participants. The final sample included 39 assault survivors. Finally, for the exploratory multiple linear regression analyses examining the association between PTSD symptom dimensions and SF impairment among survivors, an a priori power analysis was conducted using a fixed model (*R* ^2^ deviation from zero). To improve model parsimony and reduce the risk of overfitting given the available sample size, the regression models were restricted to two predictors (hyperarousal and negative alterations in cognition and mood). Assuming *α* = 0.05, power = 0.80, and a large effect size (*f* ^2^ = 0.35), the estimated required sample size was 31 participants. The final survivor sample included 39 participants.

The institutional ethics committee approved the study, and all participants provided written informed consent before participation.

### 2.2. Materials

#### 2.2.1. PTSD Symptom Severity and Clusters

PTSD symptoms were assessed using the Clinician‐Administered PTSD Scale for DSM‐5—past month version (CAPS‐5; [36]), which evaluates 20 PTSD symptoms on a 5‐point Likert scale ranging from 0 (no impairment) to 4 (extreme impairment). Total PTSD severity was calculated by summing all item scores (range: 0–80), with higher scores indicating greater symptom severity. The total CAPS‐5 score ranges from 0 to 80, with higher scores indicating greater PTSD severity. Symptom clusters of PTSD were also assessed with the CAPS‐5 according to the DSM‐5: Criterion B (reexperiencing) includes 5 items (range: 0–20), Criterion C (avoidance) includes two items (range: 0–8), Criterion D (negative alterations in cognition and mood) includes 7 items (range: 0–28), and Criterion E (hyperarousal) includes 5 items (range: 0–24); additionally, dissociation was assessed by summing two items. Criterion F requires that the disturbance has lasted at least 1 month, and Criterion G requires that the disturbance cause either clinically significant distress or functional impairment. PTSD diagnosis was established according to DSM‐5 criteria, requiring the presence of the minimum symptom thresholds for Criteria B–E, together with a symptom duration of at least 1 month (Criterion F) and clinically significant distress or functional impairment (Criterion G). The CAPS‐5 has demonstrated high internal consistency (*α* = 0.88) and test–retest reliability (*κ* = 0.83) [36]. In the present sample, the total CAPS‐5 score showed excellent internal consistency (Cronbach’s *α* = 0.90). Internal consistency was acceptable for the intrusion (*α* = 0.74), negative alterations in cognition and mood (*α* = 0.72), hyperarousal (*α* = 0.76), and functional impairment (*α* = 0.73) clusters, whereas the avoidance cluster showed a lower Cronbach’s *α* coefficient (*α* = −0.152). This lower coefficient was expected, as this cluster is composed of only two items, reflecting the well‐recognized dependence of *α* on scale length rather than a necessarily poorer measurement quality.

#### 2.2.2. Changes in SF

SF was assessed in survivors and controls using the Spanish version of the Changes in SF Questionnaire (CSFQ), a validated instrument designed to evaluate changes in SF following clinically relevant events, such as medication use or stressful life experiences [37]. The female version of the CSFQ (34 items) was exclusively administered, as it was the most appropriate for our all‐female sample.

The CSFQ follows DSM‐IV criteria for sexual dysfunction and includes two assessment conditions: a measure of baseline SF and an assessment of current SF after a significant event. For the baseline condition, participants were instructed: “*Please rate your usual sexual functioning before the sexual assault, considering your typical experiences before the event*.” For the postassault condition, they were instructed: “*Please rate your current sexual functioning following the traumatic event, reflecting your experiences after the stressor*.”

The CSFQ evaluates five key dimensions of SF through 12 items: sexual desire/frequency, sexual desire/interest, sexual pleasure, arousal/excitement, and orgasm/completion. Responses are recorded on a 5‐point Likert scale, measuring frequency (“never” to “every day”), satisfaction (“none” to “great”), or change (“no change” to “greatly improved”). In accordance with the study aims and to reduce multiple testing, analyses focused on the global CSFQ score, calculated by summing the domain scores together with two additional items assessing the loss of arousal and painful orgasm.

The CSFQ has demonstrated strong psychometric properties, with high internal consistency (*α* = 0.80) and test–retest reliability (*κ* = 0.85) [38]. In the present sample, internal consistency was good for the baseline assessment (Cronbach’s *α* = 0.79) and excellent for the postassault assessment (Cronbach’s *α* = 0.94).

### 2.3. Procedure

Potentially eligible participants were identified through the review of clinical records and subsequently contacted by telephone by members of the clinical team, who provided information about the study and invited them to participate. Participation was entirely voluntary, and no financial or other incentives were offered. Refusal or acceptance to participate had no impact on access to or continuity of clinical care.

Participants who agreed to participate attended a single in‐person assessment lasting ~90 min at the Foundation, where survivors had previously received specialized support. After providing written informed consent, participants completed the study assessments in a quiet private room. Sociodemographic information was obtained from a combination of clinical records and a brief structured interview conducted during the assessment session. Information on assault characteristics, including the method of coercion and other contextual variables, was extracted primarily from clinical and police records to minimize unnecessary recollection of the traumatic event. When relevant information was unavailable or incomplete, brief semi‐structured interviews were conducted to obtain the missing details.

Clinical psychologists experienced in trauma assessment, standardized psychological measures, and semi‐structured clinical interviews conducted the assessments, all working within a specialized sexual trauma service. Notably, interviewers had not previously worked with the participants, ensuring that the evaluations were independent of any prior therapeutic relationship.

Survivors (DFSA and non‐DFSA groups) completed the CAPS‐5 together with both CSFQ assessments, evaluating retrospective pre‐assault and current postassault SF. Control participants completed only the baseline CSFQ assessment, as the study was designed to evaluate trauma‐related changes in SF following a recent SA. Because control participants had not experienced a comparable index event, repeated assessment was conducted only with the survivors’ data.

### 2.4. Data Analysis

Continuous variables were presented as means and standard deviations, whereas categorical variables were reported as frequencies and percentages. Group differences in sociodemographic characteristics were examined using one‐way ANOVA for continuous variables, or Kruskal–Wallis tests when distributional assumptions were not met, and chi‐square tests for categorical variables.

Analyses were conducted in three stages corresponding to the primary, secondary, and exploratory study objectives. First, to address the primary objective, PTSD symptomatology was compared between DFSA and non‐DFSA survivors. Because CAPS‐5 symptom dimensions are conceptually and empirically interrelated, two between‐group MANOVAs were conducted to examine group differences in symptom count and symptom severity across the six CAPS‐5 dimensions (intrusion, avoidance, negative alterations in cognition and mood, hyperarousal, dissociation, and functional impairment). Significant multivariate effects were followed by univariate analyses. In addition, mixed ANOVAs were conducted to compare the overall PTSD symptom count and severity between groups, and chi‐square tests were used to examine group differences in PTSD diagnosis and dissociative symptomatology.

Second, to address the secondary objective, changes in SF following SA were examined using mixed repeated‐measures ANOVAs, including only the DFSA and non‐DFSA groups. To reduce multiple testing and improve parsimony, analyses focused on a global index of SF impairment rather than domain‐specific SF outcomes. Time (pre‐assault vs. postassault) was included as the within‐subject factor and Group (DFSA vs. non‐DFSA) as the between‐subject factor. Baseline SF levels for the three groups (DFSA, non‐DFSA, and control) were additionally reported descriptively to provide a reference for pre‐assault SF (see Table 1).

**Table 1:** CSFQ scale global SF at baseline and follow‐up level for non‐DFSA, DFSA survivors, and the control group.

	Time of assessment	Non‐DFSA (*N* = 20)	DFSA (*N* = 19)	Control (*N* = 35)
Global SF	Baseline	41.20 (7.38)	44.32 (6.22)	44.28 (4.48)
	Follow‐up	27.55 (10.6)	30.58 (11.09)	—

*Note:* Data are presented as means (M), with standard deviations (SD) shown in parentheses.

Abbreviations: DFSA, drug‐facilitated sexual assault; SF, sexual functioning.

Finally, exploratory multiple linear regression analyses were conducted to examine the association between PTSD symptom dimensions and SF impairment among survivors. SF change scores were calculated by subtracting pre‐assault scores from postassault scores. Given the modest sample size, the regression strategy was simplified to improve parsimony and reduce the risk of overfitting. Before model estimation, intercorrelations among CAPS‐5 symptom dimensions and their bivariate associations with SF impairment were examined to assess potential multicollinearity and guide predictor selection. Because the CAPS‐5 symptom dimensions showed substantial overlap, only symptom clusters demonstrating both theoretical relevance and the strongest associations with SF impairment were retained. Specifically, hyperarousal symptoms and negative alterations in cognition and mood were included in the final model.

All statistical analyses were conducted using Jamovi (version 2.6). Data, analytic code, and study materials are available from the corresponding author upon reasonable request.

## 3. Results

The sociodemographic and assault‐related characteristics are presented in Table 2. The groups were comparable in sex, age, educational level, and nationality (all *ps* > 0.069). Participants ranged in age from 18 to 46 years (M = 26,3, SD = 7.06). Within the DFSA group, 31.6% reported voluntary substance intake, 15.8% involuntary intake, and 53.6% mixed intake, most commonly involving alcohol combined with other substances. Comparisons between the two survivor groups showed no significant differences in assault characteristics, including assault type, time elapsed since the assault, and relationship with the perpetrator (all *ps* > 0.13). As expected, DFSA survivors reported significantly greater memory impairment (*p* = 0.037), with a higher proportion experiencing complete or partial amnesia for the assault.

**Table 2:** Sociodemographic details for non‐DFSA survivors, DFSA survivors, and the control group.

	Non‐DFSA (*N* = 20)	DFSA (*N* = 19)	Control (*N* = 35)	*p*
Females (*n*)	20	19	35	—
Age	25.3 (7.29)	30.2 (8.81)	24.8 (5.17)	0.069
Education (*n*)	—	—	—	0.172
Primary studies	3	1	0	—
Secondary studies	6	5	14	—
University studies	11	13	21	—
Nationality (*n*)	—	—	—	0.087
European	15 (75%)	12 (63.2%)	31 (88.6%)	—
Latin American	5 (25%)	7 (6.8%)	4 (11.4%)	—
Sexual orientation (*n*)	—	—	—	0.778
Heterosexual	16 (80%)	15 (78.9%)	30 (85.7%)	—
Homosexual	0	0	0	—
Bisexual	4 (20%)	4 (21%)	5 (14.3%)	—
Religion (*n*)	—	—	—	0.404
Believer	9 (45%)	10 (52.6%)	12 (34.3%)	—
Nonbeliever	11 (55%)	9 (47.4%)	23 (65%)	—
Marital status (*n*)	—	—	—	0.523
With a partner	8 (40%)	7 (36.8%)	18 (51.4%)	—
Without a partner	12 (60%)	12 (63.2%)	17 (48.6%)	—
Type of SA (*n*)	—	—	—	0.2
With penetration	8 (40%)	15 (78.9%)	—	—
Without penetration	12 (60%)	4 (21.1%)	—	—
Time since the SA	10.10 (7.23)	8.58 (5.75)	—	0.475
Injuries after SA (*n*)	—	—	—	0.345
Severe (fractures, tears)	4 (20%)	2 (10.5%)	—	—
Mild (contusions)	9 (45%)	6 (31.6%)	—	—
No injuries	7 (35%)	11 (57.9%)	—	—
Type of intake (*n*)	—	—	—	**<0.001**
Voluntary intake	0	6 (31.6%)	—	—
Involuntary intake	0	3 (15.8%)	—	—
Mixed intake	0	10 (53.6%)	—	—
No intake	20 (100%)	0	—	—
Substance ingested	—	—	—	—
Alcohol	—	7 (36.8%)	—	**<0.001**
Other substances (drugs or medication)	—	3 (15.8%)	—	—
Mixed substances	—	9 (47.4%)	—	—
Report (*n*)	16 (80%)	15 (78.9%)	—	0.935
Pregnancy (*n*)	0	0	—	—
Memory of the assault	—	—	—	**0.037**
No memories	3 (15%)	8 (42.1%)	—	—
Partial memories	5 (25%)	7 (36.8%)	—	—
Full memory	12 (60%)	4 (21.1%)	—	—
Number of therapy sessions	4.4 (4.45)	2.36 (5.49)	—	**<0.001**
Number of perpetrators	1 (0.00)	1.15 (0.37)	—	**<0.001**
Age of perpetrators	32.35 (13.56)	28.84 (14.69)	—	**<0.001**
Nationality of perpetrators (*n*)	—	—	—	0.15
Same as the survivor	14 (70%)	6 (31.6%)	—	—
Different from the survivor	6 (30%)	13 (68.4%)	—	—
Male perpetrators (*n*)	20 (100%)	19 (100%)	—	—
Relationship with the perpetrator (*n*)	—	—	—	0.13
Stranger	4 (20%)	5 (26.3%)	—	—
Recent acquaintance	7 (35%)	11 (57.9%)	—	—
Intimate relationship	9 (45%)	3 (15.8%)	—	—
Scenario of the SA (*n*)	—	—	—	0.33
Private	13 (65%)	15 (78.9%)	—	—
Public	7 (35%)	4 (21.1%)	—	—
Perceived psychological consequences (*n*)	16 (80%)	16 (84.2%)	—	0.73
Physical consequences (*n*)	11 (55%)	8 (42.1%)	—	0.42
Sexually transmitted infections (*n*)	5 (25%)	3 (15.8%)	—	0.47

*Note:* Data are presented as frequency (*N*) or means (M), with standard deviations (SD) shown in parentheses. Significant *p* values are shown in bold.

Abbreviations: DFSA, drug‐facilitated sexual assault; SA, sexual assault.

### 3.1. Number and Severity of Each PTSD Symptom Cluster

Table 3 summarizes the number and severity of PTSD symptom clusters in the two survivor groups. As illustrated in Figure 2a, the MANOVA on symptom count showed a significant multivariate group effect, *F* (6, 32) = 3.75, *p* = 0.006; Pillai’s trace = 0.413. Follow‐up univariate analyses indicated that survivors of non‐DFSA reported a greater number of reexperiencing symptoms, *F* (1, 37) = 6.73, *p* = 0.013, *η* ^2^ = 0.154, distress or impairment, *F* (1, 37) = 4.90, *p* = 0.033, *η* ^2^ = 0.117, and dissociation, *F* (1, 37) = 12.017, *p* = 0.001, *η* ^2^ = 0.245, than DFSA survivors. No significant group differences were observed for avoidance, negative alterations in cognition and mood, or hyperarousal symptoms (all *ps* > 0.24).

**Figure 2:**
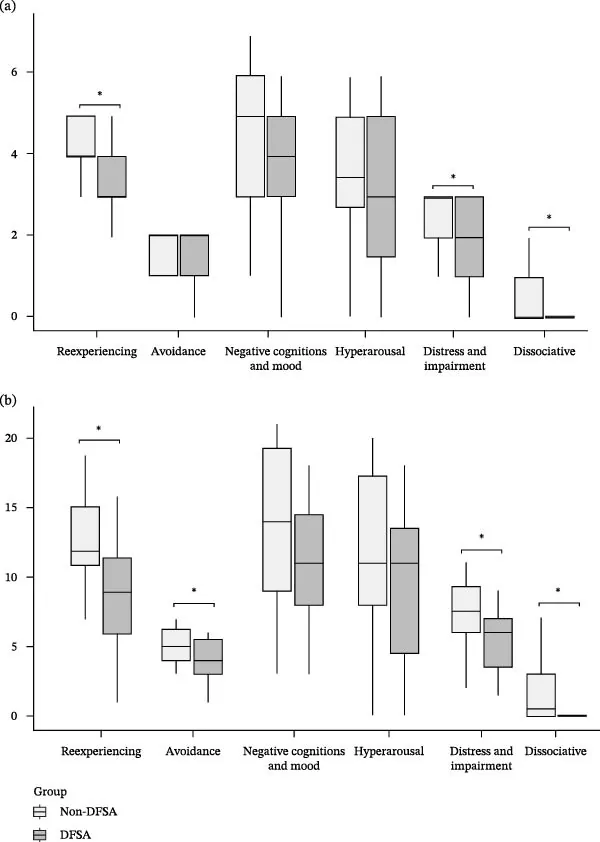
Box plots showing the mean number (a) and severity (b) of symptoms on the CAPS‐5 scale for the non‐DFSA and DFSA groups. Bars represent mean values; error bars indicate the standard error of the mean. Statistically significant differences (*p* < 0.05) are denoted by an asterisk ( ^∗^).

**Table 3:** CAPS‐5 scale results for non‐DFSA and DFSA survivors.

	Non‐DFSA (*N* = 20)	DFSA (*N* = 19)	*p*	*η* ^2^
Number of symptoms				
Reexperiencing	**4.10 (0.85)**	**3.05 (1.58)**	**0.013**	**0.154**
Avoidance	1.70 (0.47)	1.47 (0.70)	0.240	—
Negative cognition and mood	4.35 (1.84)	3.79 (1.58)	0.316	—
Hyperarousal	3.50 (1.70)	3.10 (1.94)	0.503	—
Distress	**2.60 (0.68)**	**1.89 (1.24)**	**0.033**	**0.117**
Dissociative	**0.60 (0.75)**	**0.00 (0.00)**	**0.001**	**0.245**
Severity				
Reexperiencing	**12.65 (3.30)**	**8.84 (4.39)**	**0.004**	**0.203**
Avoidance	**5.20 (1.36)**	**4.21 (1.51)**	**0.038**	**0.111**
Negative cognition and mood	13.70 (6.04)	11.31 (4.57)	0.175	—
Hyperarousal	11.75 (5.63)	9.52 (5.71)	0.228	—
Distress	**7.55 (2.48)**	**5.26 (2.60)**	**0.008**	**0.176**
Dissociative	**1.70 (2.10)**	**0.05 (0.23)**	**0.002**	**0.237**
Total number of symptoms	13.65 (3.53)	11.42 (4.42)	0.090	—
Total severity	**43.30 (13.21)**	**33.89 (12.72)**	**0.030**	**0.122**

*Note*: Data are presented as means (M), with standard deviations (SD) shown in parentheses. Significant *p* values are shown in bold. CAPS‐5, Clinician‐Administered PTSD Scale for DSM‐5.

Abbreviations: DFSA, drug‐facilitated sexual assault; PTSD, posttraumatic stress disorder.

Similarly, the MANOVA on symptom severity revealed a significant multivariate group effect, *F* (6, 32) = 3.31, *p* = 0.012; Pillai’s trace = 0.383 (Figure 2b). Compared with DFSA survivors, the non‐DFSA group exhibited higher severity in reexperiencing (*F*(1, 37) = 9.45, *p* = 0.004, *η* ^2^ = 0.203), avoidance behaviors (*F*(1, 37) = 4.62, *p* = 0.038, *η* ^2^ = 0.111), distress or impairment (*F*(1, 37) = 7.90, *p* = 0.008, *η* ^2^ = 0.176), and dissociation (*F*(1, 37) = 11.49, *p* = 0.002, *η* ^2^ = 0.237). No significant between‐group differences emerged for hyperarousal or negative alterations in cognition and mood (both *ps* > 0.17).

### 3.2. Presence of PTSD

Fourteen non‐DFSA survivors and nine DFSA survivors met the PTSD criteria. However, the chi‐square test revealed no significant differences between survivor groups, *χ* ^2^(1) = 2.06, *p* = 0.15.

### 3.3. Global SF After SA

At baseline, global SF did not differ significantly across the three groups (DFSA, non‐DFSA, and control; *p* = 0.144; see Table 1).

Among SA survivors, the mixed repeated‐measures ANOVA revealed a significant main effect of Time, *F*(1, 37) = 54.30, *p* < 0.001, *η* ^2^ = 0.368, indicating a significant change in global SF from pre‐assault to postassault assessment. The main effect of Group was not significant, *F*(1, 37) = 1.89, *p* = 0.177, *η* ^2^ = 0.019. The Group × Time interaction was also not significant, *F*(1, 37) < 1, indicating that the change in global SF over time did not differ between DFSA and non‐DFSA survivors. These findings are illustrated in Figure 3. Analyses of the individual SF domains yielded a comparable pattern of results and are presented in the Supporting Information.

**Figure 3:**
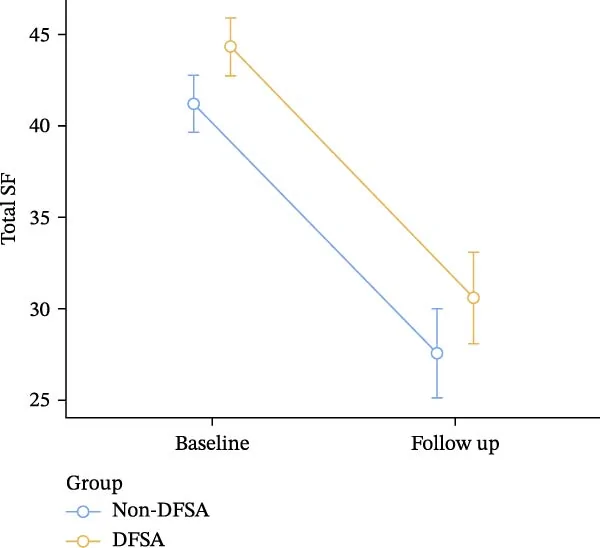
Graphs showing global sexual functioning pre‐ and post‐SA for the non‐DFSA and DFSA groups.

### 3.4. PTSD Symptom Predictors of Global SF Deterioration in SA Survivors

An exploratory multiple linear regression analysis was conducted to examine whether hyperarousal symptoms and negative alterations in cognition and mood were associated with global SF deterioration among SA survivors. The overall model was statistically significant, *F*(2, 36) = 4.46, *p* = 0.019, explaining 20% of the variance in global SF deterioration (*R* ^2^ = 0.20, adjusted *R* ^2^ = 0.154). Greater hyperarousal symptom severity was significantly associated with greater deterioration in global SF (*B* = 0.73, SE = 0.34, *t* = 2.16, *p* = 0.037). In contrast, negative alterations in cognition and mood were not significantly associated with global SF deterioration after accounting for hyperarousal symptoms (*B* = 0.29, SE = 0.35, *t* = 0.82, *p* = 0.42).

## 4. Discussion

The primary aim of this study was to characterize posttraumatic symptomatology in DFSA and non‐DFSA. Although both groups met DSM‐5 criteria for PTSD at comparable rates, DFSA survivors exhibited a distinct symptom profile, characterized by lower severity of reexperiencing, avoidance, dissociative symptoms, and functional impairment, as well as fewer symptoms within the reexperiencing, dissociation, and functional impairment clusters. Consistent with our secondary objective, both survivor groups experienced a marked decline in SF following the assault, with no differences according to the assault type. Finally, exploratory analyses indicated that greater hyperarousal symptom severity was associated with more pronounced SF deterioration, whereas negative alterations in cognition and mood were not. Together, these findings suggest that although DFSA and non‐DFSA survivors experience comparable overall postassault burden, drug facilitation may influence the expression of PTSD symptoms without modifying the magnitude of SF impairment.

Regarding PTSD, both DFSA and non‐DFSA survivors met PTSD criteria at comparable rates, consistent with previous studies reporting no diagnostic differences between assault types [7, 8, 15]. However, examining PTSD dimensionally revealed a more differentiated pattern. Consistent with Fields et al. [7], who also studied a clinically characterized sample with careful control of previous trauma exposure and substance use disorders, DFSA survivors exhibited lower dissociative symptom severity than non‐DFSA survivors. Extending these findings, our study also identified lower reexperiencing, avoidance, and functional impairment in the DFSA group. This broader pattern of cluster‐specific differences may reflect the more stringent methodological approach adopted, including the recruitment of survivors of a single recent assault and the exclusion of previous trauma and any pre‐existing psychiatric disorders, thereby reducing clinical heterogeneity and facilitating the identification of differential PTSD symptom profiles associated with DFSA and non‐DFSA.

A plausible explanation for these cluster‐specific differences relates to the distinctive way in which traumatic experiences are encoded during DFSA. Substance‐induced alterations in consciousness may disrupt the encoding and consolidation of the assault, resulting in fragmented or incomplete trauma memories and a less coherent autobiographical narrative [6, 7]. Such disruptions may reduce the sensory vividness and accessibility of trauma memories, thereby attenuating reexperiencing symptoms and the subsequent need to avoid trauma‐related cues. Similarly, lower functional impairment may likewise reflect reduced conscious threat processing during the assault [9], resulting in fewer trauma‐related intrusions and a less persistent perception of threat in everyday life. By contrast, the absence of group differences in negative alterations in cognition and mood suggests that differences in trauma encoding may influence the qualitative content of posttraumatic appraisals more than their overall severity. While survivors of non‐DFSA may predominantly develop fear‐ and coercion‐related interpretations of the assault, DFSA survivors may be more likely to experience confusion, self‐blame associated with substance use, and a diminished sense of control [10, 11]. Although these cognitive themes differ, they may generate comparable levels of emotional distress and negative self‐perception, resulting in similar scores on this PTSD symptom dimension. Similarly, the absence of group differences in hyperarousal suggests that persistent physiological activation may represent a fundamental response to sexual victimization, regardless of whether the assault involved drug facilitation.

Regarding SF, and consistent with our hypothesis, both DFSA and non‐DFSA survivors showed a marked decline in overall SF following the assault, with a similar pattern observed across all individual SF domains (see Supporting Information). No differences were found between the two survivor groups. These findings reinforce the well‐established association between SA and subsequent sexual difficulties [22–24] and extend previous evidence by demonstrating that this deterioration is also present following DFSA. Together with the PTSD findings, these results suggest that although DFSA survivors display a distinct posttraumatic symptom profile, the overall impact of SA on SF appears comparable across assault types. This convergence indicates that different trauma‐processing pathways may ultimately lead to similar levels of sexual impairment, highlighting SF difficulties as a common consequence of sexual victimization, regardless of whether drug facilitation was involved.

Finally, the exploratory analyses provide preliminary evidence regarding the mechanisms that may underlie this deterioration in SF. Unlike previous studies that conceptualized PTSD categorically [3], we examined specific PTSD symptom dimensions and found that hyperarousal was the only independent predictor of global SF deterioration. This finding is consistent with theoretical models proposing PTSD as a key mechanism linking SA and later sexual difficulties [32, 33] and extends previous evidence from female veteran samples [34] to a clinically homogeneous sample of survivors without previous trauma exposure or psychiatric disorders. Hyperarousal reflects persistent physiological activation and impaired regulation of autonomic responses [35], processes that may interfere with SF by maintaining vigilance, increasing somatic tension, and reducing the capacity to engage in intimate experiences [33]. Contrary to our hypothesis, negative alterations in cognition and mood did not independently predict overall SF deterioration. A possible explanation is that hyperarousal represents a more proximal mechanism linking PTSD to SF impairment, whereas cognitive‐affective symptoms may have a more specific or indirect influence that is not fully captured by a global SF measure. These findings should be interpreted cautiously given the exploratory nature of the analyses, but they suggest that interventions targeting physiological hyperarousal may represent a promising avenue for improving sexual recovery after SA.

Despite the relevance of the findings, several limitations should be considered. First, the relatively small sample size, resulting from the stringent inclusion and exclusion criteria, may have limited statistical power, the complexity of the regression analyses, and the generalizability of the findings, particularly given the high prevalence of revictimization among SA survivors. Nevertheless, these restrictive criteria strengthened the study’s internal validity by enabling us to examine the psychological consequences of a single recent SA while minimizing the potential influence of previous trauma and pre‐existing psychiatric conditions. However, the small sample size may also have affected the stability of the reliability estimates. In particular, the CAPS‐5 avoidance cluster showed low internal consistency in our sample (*α* = −0.152), with its two items displaying a near‐zero inter‐item correlation (*r* = −0.044). Therefore, the statistically significant group difference in avoidance symptom severity should be interpreted cautiously and considered preliminary. Second, although classifying survivors into DFSA and non‐DFSA groups enabled the specific examination of drug‐facilitated assaults, this categorization inevitably simplifies the complexity of SA experiences, which frequently involve multiple overlapping coercive strategies (e.g., physical force, psychological coercion, threats, and substance use). Accordingly, the present classification should be viewed as an operational framework rather than as representing mutually exclusive assault types. Future studies should examine more diverse assault profiles to better capture this complexity. Third, SF was assessed through self‐report, which may be influenced by recall or social desirability biases. Nevertheless, the CSFQ remains a standardized instrument specifically designed to detect changes in SF across multiple domains and was therefore well suited to the aims of the present study despite being originally developed under DSM‐IV criteria. Finally, control participants completed only a baseline SF assessment. Consequently, the study was not designed to examine natural temporal changes in SF among women without SA, and it did not provide direct longitudinal comparisons with the survivor groups.

Despite these limitations, the present study has several important strengths. To our knowledge, it is among the first to directly compare PTSD symptom profiles and SF in DFSA and non‐DFSA survivors using clinically well‐characterized groups of women who experienced a single recent SA. By applying stringent inclusion criteria, we minimized the influence of major confounding factors, thereby strengthening the internal validity of the study and enabling a more specific examination of the psychological consequences attributable to the index assault. In addition, the inclusion of both a non‐DFSA survivor group and a matched nontraumatized control group provided clinically meaningful comparison groups for interpreting PTSD symptoms and the magnitude of postassault SF impairment. Finally, rather than relying exclusively on categorical PTSD diagnosis or overall symptom severity, the present study adopted a dimensional approach by examining individual PTSD symptom clusters and their relationship with SF. This strategy enabled the identification of clinically meaningful differences that would have remained undetected using global PTSD scores alone, providing a more nuanced characterization of posttraumatic responses following DFSA and non‐DFSA and extending current knowledge regarding the specific symptom dimensions associated with postassault SF.

## 5. Conclusions

This study provides further evidence that DFSA is associated with a distinct pattern of PTSD symptom expression despite comparable rates of PTSD diagnosis and similar deterioration in SF relative to non‐DFSA survivors. These findings may provide a more nuanced understanding of the psychological consequences of different forms of SA. Regarding clinical implications, our findings support the systematic assessment of individual PTSD symptom clusters in survivors of SA. Although DFSA survivors exhibited lower reexperiencing, avoidance, dissociative symptoms, and functional impairment, hyperarousal emerged as the only independent predictor of global SF deterioration across all survivors. This suggests that persistent physiological activation may represent a common therapeutic target when addressing postassault sexual difficulties, regardless of whether the assault involved drug facilitation. Accordingly, interventions aimed at reducing autonomic hyperactivation and persistent defensive responding may contribute not only to PTSD recovery but also to improving SF after SA.

Future research should move beyond the categorical distinction between DFSA and non‐DFSA by incorporating dimensional indicators such as the degree of incapacitation, extent of memory impairment, disruption of the trauma narrative, and type of substance exposure. Such approaches may provide a more comprehensive understanding of PTSD symptom profiles and ultimately inform more targeted interventions for survivors of SA. Building on the exploratory findings of the present study, future work should replicate and extend these results by investigating the psychological mechanisms linking PTSD symptom dimensions and SF following SA. Larger longitudinal studies are needed to determine whether trauma‐related cognitions, emotional responses, and hyperarousal differentially contribute to SF over the course of recovery and whether these associations vary according to the assault characteristics. Finally, intervention studies should examine whether treatments targeting hyperarousal and autonomic regulation improve both PTSD symptoms and SF following SA.

## Author Contributions

**Marta Lizarán**: conceptualization, data curation, funding acquisition, investigation, methodology, resources, software, supervision, validation, visualization, writing – original draft, writing – review and editing. **Adrián Torres-Villasante**, **Julia Andreu-Martínez, Alba Moreno-Giménez, and Javier Plumed-Domingo**: funding acquisition, investigation. **Nadina Gómez-Merino**: data curation, investigation, methodology, software. **Javier Sánchez-Ruiz**: formal analysis, funding acquisition, methodology. **Ana García-Blanco**: conceptualization, formal analysis, funding acquisition, investigation, project administration, supervision, validation, writing – review and editing.

## Funding

This work was supported by the Carlos III Health Institute, co‐founded by European Union (Grants CM22/00097, PI21/00549, PI24/01259, CP21/00085, CM24/00123, and PMP22/00088), the NextGenerationEU through (Grant MS21‐063), and the Generalitat Valenciana (Grants CIAICO/2022/233 and CIACIF/2024/002).

## Ethics Statement

This study was approved by the institutional committee (Ethics Code 2020‐065‐1) on May 19, 2020. All participants provided written informed consent prior to enrollment in the study.

## Conflicts of Interest

The authors declare no conflicts of interest.

## Supporting Information

Additional supporting information can be found online in the Supporting Information section.

## Supporting information


**Supporting Information** The Supporting Information presents the complete analyses of the individual domains of sexual functioning assessed with the CSFQ (Desire/Frequency, Desire/Interest, Pleasure, Arousal, and Orgasm), reporting the mixed‐design ANOVA results. Table S1 summarizes the descriptive statistics (means and standard deviations) for each sexual functioning domain at baseline and follow‐up in both survivor groups.

## Data Availability

The data, materials, and analysis codes used in this study are not publicly available due to the sensitive nature of the information. However, they can be accessed upon reasonable request from the corresponding author, in accordance with ethical and confidentiality guidelines.
