# Supplementary material for: A Dimensional Approach to PTSD After Drug‐Facilitated Sexual Assault: Impact on Sexual Functioning
Source: Depress Anxiety. 2026 Oct 31;2026:6023533. doi: 10.1155/da/6023533 (PMC13723313; doi:10.1155/da/6023533)
Supplement: Supplementary file 1 [file DA-2026-6023533-s001.docx]

**Supplementary material**

This supplementary file presents the complete analyses of the individual domains of sexual functioning assessed with the CSFQ (Desire/Frequency, Desire/Interest, Pleasure, Arousal, and Orgasm). The subsequent sections report the mixed-design ANOVA results. Table S1 summarizes the descriptive statistics (means and standard deviations) for each sexual functioning domain at baseline and follow-up in both survivor groups. These analyses complement the main manuscript by providing the domain-specific findings underlying the global sexual functioning outcome.

**Sexual Functioning Subscales Analyses**

#### 1.1. **Desire and Frequency**

The mixed repeated-measures ANOVA revealed a significant main effect of Time, F(1, 37) = 46.97, p < .001, η² = .328. However, no significant effect of Group, F(1, 37) = 2.55, p < .119, η² =.027 nor the Group **×** Time interaction, F(1, 37) < 1were found.

#### 1.2. **Desire and Interest**

The mixed repeated-measures ANOVA revealed a significant main effect for Time, F(1, 37) = 39.82, p < .001, η² = .283. Neither Group, F(1, 37) < 1, nor the Group × Time interaction were significant, F(1, 37) = < 1.

### *****1.3. Pleasure*****

The mixed repeated-measures ANOVA revealed a significant main effect for Time, F(1, 37) = 93.52, p < .001, η² = .497. The Group, F(1, 37) < 1, and the Group × Time interaction were no significant, F(1, 37) < 1.

### *****1.4. Arousal*****

The mixed repeated-measures ANOVA revealed a significant main effect of Time, F(1, 37) = 40.69, p < .001, η² = .271. The Group, F(1, 37) < 1, and the Group × Time interaction were no significant, F(1, 37) < 1.

### *****1.5. Orgasm*****

The mixed repeated-measures ANOVA revealed a significant main effect of Time, F(1, 37) = 31.54, p < .001, η² = .229. Neither the Group, F(1, 37) = 2.42, p = .128, η² = .031, nor the Group × Time interaction were significant, F(1, 37) < 1.

Table S1 shows the means of baseline and follow-up levels of each SF domain for each survivor group.

**Supplementary Table S1**

*CSFQ scale results at the baseline and follow-up level for each SF domain in non-DFSA and DFSA survivors*

|  | Time of Assesment | Non-DFSA (N=20) | DFSA (N=19) |
| --- | --- | --- | --- |
| Desire and Frequency | Baseline | 6.70 (1.42) | 7.16 (1.30) |
|  | Follow-Up | 4.20 (1.70) | 5.05 (2.09) |
| Desire and Interest | Baseline | 8.20 (2.19) | 8.47 (2.29) |
|  | Follow-Up | 5.10 (2.22) | 5.95 (2.39) |
| Pleasure | Baseline | 3.75 (0.91) | 4.05 (0.62) |
|  | Follow-Up | 2.00 (1.03) | 2.11 (1.15) |
| Arousal | Baseline | 9.50 (2.69) | 10.16 (2.27) |
|  | Follow-Up | 6.25 (3.06) | 6.68 (3.16) |
| Orgasm | Baseline | 9.15 (2.74) | 10.47 (2.46) |
|  | Follow-Up | 6.05 (3.15) | 7.11 (3.49) |

DFSA = drug-facilitated sexual assault; CSFQ = Changes in Sexual Functioning Questionnaire

*Note*. Data are presented as means (M), with standard deviations (SD) shown in parentheses.

The supplementary figure present visualizations of sexual functioning changes across its different domains.

**Supplementary Figure S1. Changes in sexual functioning following sexual assault**

*Note.* Plots showing changes in scores across the different domains of sexual functioning from before to after the sexual assault among the survivor groups.
